# Effects of Bandwidth on Ear Differentiation and Grain Yield Formation of Maize in Strip Intercropping

**DOI:** 10.3390/plants14071081

**Published:** 2025-04-01

**Authors:** Bing Liang, Jingjing Li, Xuyang Zhao, Xinhui Lei, Guopeng Chen, Tian Pu, Yushan Wu, Taiwen Yong, Feng Yang, Xiaochun Wang, Wenyu Yang

**Affiliations:** 1 College of Agronomy, Sichuan Agricultural University, 211-Huimin Road, Wenjiang District, Chengdu 611130, China; liangbing92@163.com (B.L.); jingjingli2025@163.com (J.L.); zhao_xuyang@stu.sicau.edu.cn (X.Z.); leixinhui2020@163.com (X.L.); 2018101009@stu.sicau.edu.cn (G.C.); tianpu@sicau.edu.cn (T.P.); yushan.wu@sicau.edu.cn (Y.W.); yongtaiwen@sicau.edu.cn (T.Y.); f.yang@sicau.edu.cn (F.Y.); mssiyangwy@sicau.edu.cn (W.Y.); 2 Sichuan Engineering Research Center for Crop Strip Intercropping System, Key Laboratory of Crop Ecophysiology and Farming System in Southwest China (Ministry of Agriculture), Chengdu 611130, China

**Keywords:** intercropping, bandwidth, maize, ear development, yield

## Abstract

In strip intercropping, increasing bandwidth enhances light energy utilization and facilitates mechanized production, yet it constrains the realization of maize yield advantages. The impact of bandwidth on the ear differentiation and development and yield formation requires further investigation. In this study, different bandwidths (T1, 1.6 m, T2, 2.0 m, T3, 2.4 m, and T4, 2.8 m) were arranged, and monoculture maize with varying row spacings (K1, 0.8 m, K2, 1.0 m, K3, 1.2 m, and K4, 1.4 m) was used as the control. The results show that increasing bandwidth inhibited the ear differentiation. The proportion of dry matter partitioning to leaves increased and to ears decreased, resulting in shorter ear length and higher floret and grain abortion rates. Maize yield losses amounted to 26.9% and 31.6% in T4 compared to K4 and T1, respectively. Moreover, the bandwidth did not affect the fertilized florets due to the smaller anthesis–silking interval created by the simultaneous effect. We concluded that the appropriate bandwidth, 1.6 m and 2.0 m, can stabilize the dry matter partitioning to the ear; stabilize ear length, floret, and grain abortion rate; and stabilize the maize yield.

## 1. Introduction

Maize is an important food crop and industrial raw material. Over the past five years, China’s maize imports have risen sharply, reaching an import volume of 30.5 million metric tons and a maize area of 44.26 million hectares in 2023. To meet the demand for maize in China, approximately 48.23 million hectares of cultivated land are required, and further expanding the planting area will inevitably come at the expense of other crops (FAO, update 19 December 2024). Improving the utilization rate of light energy and increasing land output has become the primary method to enhance food supply capacity. Intercropping significantly boosts the productivity of cultivated land per unit area [1,2,3]. Intercropping enhances the photosynthetic performance of leaves on the strong light side and optimizes carbohydrates partitioning to the ear, thereby stabilizing maize yield [4]. This approach also facilitates the cultivation of other crops in wider rows. In particular, maize strip intercropping effectively alleviates the conflict between maize and autumn crops in China, particularly soybeans, thereby ensuring food security [5,6].

From the jointing stage to the maturity stage, three-quarters of the maize growth period in this planting system is under a special light environment, with weak light on one side and strong light on the other [7]. Each maize row possesses a marginal advantage in this intercropping system [8,9]. The photosynthetically active radiation (PAR) on the narrow-row side increases as the maize narrow-row spacing increases, and the PAR on the wide-row side increases as the maize wide-row spacing increases in maize strip intercropping when the bandwidth is fixed. When the wide-row spacing reaches 1.6 m, the PAR on the wide-row side tends to stabilize [7]. In actual production, by adjusting row spacing, density, and fertilization levels, the photosynthetic active radiation of wide rows is increased and the canopy light distribution is improved. We have identified the optimal field configuration for Southwest China, considering resource utilization efficiency [6] and land productivity [10]. The maize row spacing is set at 0.4 m, while the combined row spacing for maize and legumes is 0.6 m, with a bandwidth of 2 m. In this field configuration, the photosynthetic performance of unshaded leaves is increased, and sugar export is enhanced, which can effectively compensate for the reduced photosynthetic performance of unshaded leaves. Therefore, intercropping maize achieves a yield equivalent to or higher than monoculture maize [4,11,12,13].

Crops often cast shade upon one another, which can limit photosynthesis [7] and reduce biomass accumulation [14]. All processes of ear differentiation and development—including floret differentiation, ear elongation, fertilization, and grain development—are affected, resulting in yield loss [15,16,17,18,19,20,21]. As the breeding process with high-density planting for high yield progresses, the maize grain yield is constrained by grains per ear and grain weight [22,23,24]. The photosynthetic performance of leaves between two sides under asymmetric light of strip intercropping maize is different, and the carbohydrates partitioning to the ear are different [4]. Grains per ear is the primary factor limiting the yield in maize strip intercropping [5]. The strong nitrogen fixation ability of legumes and the activation of soil nutrients by rhizosphere exudates can effectively improve the rate of utilization of maize nutrients, leading to a yield advantage [3,8,25]. Therefore, a large number of studies have focused on improving the photosynthetic performance of maize and the interaction effect with adjacent crops under optimal bandwidth [4,13]. Nevertheless, expanding the bandwidth benefits the light energy utilization [6] and mechanization of neighboring crops, typically legumes. Conversely, for maize, wider bandwidths, reduced plant spacing, and heightened intraspecific competition tend to undermine the crop’s yield advantage [26]. The effect of bandwidth on maize ear development under asymmetric light environment in strip intercropping has not been reported.

Thus, in this study, optimal narrow row spacing for maize was fixed, the bandwidth was varied, and consequently, the plant spacing was varied. Simultaneously, the corresponding monoculture maize with equal plant spacing as intercropping was used as the control group. The main purpose was (i) to analyze the response of each stage of ear differentiation and development to asymmetric light environments, and (ii) to access the main limiting process of ear development on maize yield formation in strip intercropping. This study enriches the theory of crop marginal advantage from the perspective of female ear development, and lays a theoretical foundation for high-yield techniques of crop cultivation.

## 2. Materials and Methods

### 2.1. Plant Materials and Field Site

**Figure 1:**
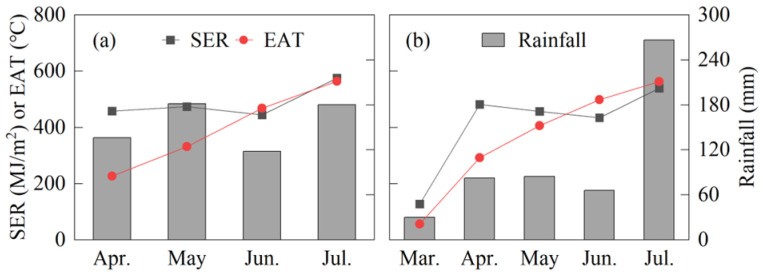
Monthly rainfall, effective accumulated temperature (EAT), and solar effective radiation (SER) during the maize growth period in 2022 (**a**) and 2023 (**b**). The data are from NASA (https://worldwind.arc.nasa.gov/worldweather/, accessed on 9 October 2024).

The maize variety “Zhongyu 3” was selected for widespread promotion and is suitable for maize intercropping. The experiment was conducted at the Chongzhou Modern Agricultural Research and Development Base (103°06′ E, 30°06′ N), located in Sichuan Province, China. The site’s altitude is 523 m, with a subtropical humid monsoon climate. The monthly rainfall, effective accumulated temperature, and solar effective radiation are shown in Figure 1. The soil type is loam, with uniform soil fertility. The basic physical and chemical properties of the topsoil were determined using a five-point sampling method: pH 6.3, organic matter 23.0 g·kg^−1^, total nitrogen 1.8 g·kg^−1^, alkali-hydrolyzed nitrogen 135.7 mg·kg^−1^, available phosphorus 19.3 mg·kg^−1^, and available potassium 101.0 mg·kg^−1^.

### 2.2. Experimental Design and Field Management

**Figure 2:**
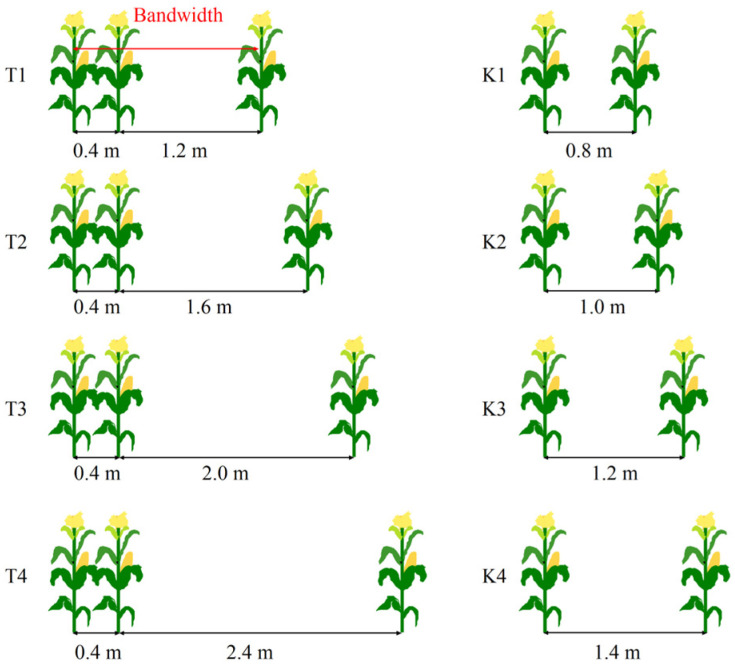
Field configurations from 2022 to 2023.

The single-factor randomized block design was employed. The factors included various bandwidths: T1: 1.6 m, T2: 2.0 m, T3: 2.4 m, and T4: 2.8 m, respectively. Controls consisted of equal planting spacing compared to corresponding intercropping: K1: 0.8 m, K1: 1.0 m, K1: 1.2 m, and K4: 1.4 m (Figure 2). A total of 8 treatments were used, each repeated 3 times, resulting in 24 plots. Each plot was planted with 3 bandwidths, with each bandwidth measuring 7 m in length. The maize density was set at 60,000 plants per hectare. The sowing took place on 30 March 2022 and 19 March 2023, with single-plant planting per hole in an east–west direction. The base fertilizer was applied at a rate of 600 kg of superphosphate (containing 12% P_2_O_5_) and 150 kg of potassium chloride (containing 60% K_2_O) per hectare. Throughout the maize growth period, 4 g pure nitrogen were applied per plant, with a base-fertilizer-to-ear-fertilizer ratio of 5:5. Other management practices were consistent with those used in the field.

### 2.3. Sampling and Measurements

#### 2.3.1. Light Transmittance of Maize Canopy

During the grain-filling stage for maize, specifically on 4 July in 2022 and 27 June in 2023 between 10:00 and 12:00 a.m., the canopy analyzer SunScan (SS1-COM Complete System, Delta-T Devices, Cambridge, UK) was used to measure the photosynthetically active radiation (PAR) of the ear layer on the wide-row and the narrow-row side. The strip intercropping maize was measured every 20 cm in the horizontal direction from the first maize row in each strip. The average PAR of the narrow row was calculated at three points, and the average PAR in the wide row was calculated at all points. In monoculture maize, we measured three points, namely, two points near the two rows of plants and the middle point of the two rows of plants. The PAR of 4 rows of maize was measured continuously in each plot, and the average PAR of ear layer of each monoculture maize was obtained. Light transmittance is the ratio of photosynthetically active radiation in the ear layer to natural unshielded photosynthetically active radiation.

#### 2.3.2. Ear Differentiation and Development

Starting from maize V7, following the method of Cui et al. [27], young ear differentiation and development were observed every 3–5 days until the silking stage. The ear differentiation period was determined using a microscope. The total florets were recorded during the differentiation period. In each plot, ten plants were continuously monitored, the onset and end time of tasseling and silking were documented, and the proportion of tasseling and silking, as well as the anthesis to silking interval (ASI), were calculated. At the end time of pollination, the bracts were carefully removed, and the silks that had fallen from the ear, as well as those that had not fallen but had withered at the base, were counted as the fertilized florets. The floret abortion rate was calculated as the ratio of the number of abortive florets (the difference between the total florets and the fertilized florets) to the total florets.

#### 2.3.3. Maize Shoot Dry Matter

At the maize silking stage, on 14 June 2022 and 4 June 2023, three representative maize plants with the same growth vigor were selected from each plot. The plants were separately packed based on the stems (except leaves and ears), leaves and ears. After being subjected to 105 °C for 1 h, they were further dried to a constant weight at 80 °C and weighed.

#### 2.3.4. Yield and Yield Components

At the maize maturity stage, specifically on 28 July 2022 and 22 July 2023, the number of production ears (NPEs) was investigated before harvest. Twenty female panicles were chosen by the average weight method in each plot. Once the ears were dried to a constant weight, measurements were taken for ear length, ear diameter, barren tip length, 1000-grain weight, and grains per ear (grains per row × row number). The grain abortion rate was expressed as the ratio of abortive grains (the difference between the fertilized florets and the grains per ear) to the fertilized florets.

### 2.4. Statistical Analysis

Statistical analysis was performed using Excel 2017 (Microsoft, Redmond, WA, USA) and SPSS 19.0 (IBM, Chicago, IL, USA). One-way analysis of variance and Duncan’s test were used to compared treatments, with *p* < 0.05 considered statistically significant. The figures were plotted by Origin 2021 (OriginLab, Northampton, MA, USA).

## 3. Results

### 3.1. Differences in Light Transmittance of Wide Row and Narrow Row

**Figure 3:**
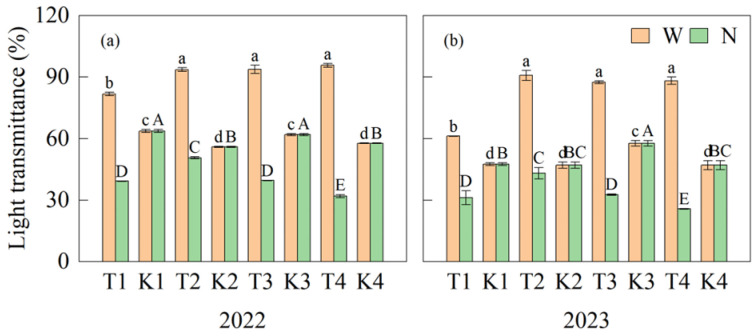
The difference in light transmittance between treatments at the grain-filling stage in 2022 (**a**) and 2023 (**b**). Different lowercase letters and capital letters above the columns represent differences at the significant level (*p* < 0.05) between treatments within the wide row (W) and the narrow row (N), respectively. Values are means (n = 3), and bars indicate standard errors.

The light transmittance of the wide-row middle layer during the grain-filling stage in strip intercropping (T treatment) was significantly higher than that of the corresponding monoculture (K treatment), while the light transmittance of the narrow row with the T treatment was significantly lower than that of the K treatment. Specifically, the average light transmittance of the ear layer of the wide row of the T treatment increased by 28.6%, 79.2%, 51.5%, and 75.7%, whereas the light transmittance of the ear layer of the narrow row decreased by 36.7%, 9.0%, 39.7%, and 45.0% over two years compared to the K treatment. As the bandwidth increased, the light transmittance of the wide row increased significantly and there was no significant difference between T2, T3, and T4, while the light transmittance in the narrow row decreased significantly. The light transmittance of the narrow row at T2 was significantly higher than that of T1, T3, and T4. The differences in light transmittance between the two sides of the maize plants with the T treatment were 36.3%, 45.4%, 54.5%, and 63.2% (Figure 3).

### 3.2. Shoot Dry Matter Accumulation and Partitioning

**Figure 4:**
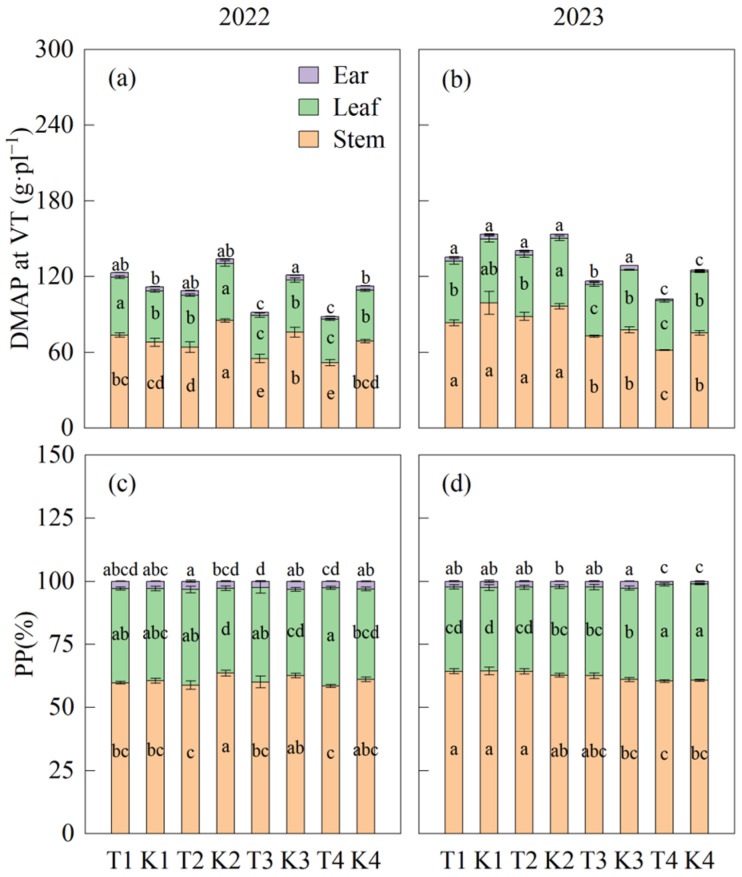
Dry matter accumulation and partitioning at the maize silking stage. (**a**,**b**) represent the dry matter accumulation and partitioning at the silking stage of maize in 2022 and 2023, respectively. (**c**,**d**) represent the dry matter partitioning proportion of each tissue during the silking stage of maize in 2022 and 2023, respectively. Different lowercase letters on the columns represent differences at the significant level (*p* < 0.05) within one plant’s tissues. Values are means (n = 3), and bars indicate standard errors.

Compared to the corresponding monoculture, there was no significant difference in the dry matter partitioning to the ear between T1 and T2 at the silking stage, while it was significantly decreased by 32.7% and 20.4% in T3 and T4, respectively. The proportion of dry matter partitioning to the ear in T3 and T4 decreased significantly. Except for T2 in 2022, which was significantly higher than K2, the proportion of partitioning to the ear showed no significant difference between T1 and K1, and T2 and K2. With bandwidth increasing, the ear dry weight decreased significantly, and there was no significant difference between T1 and T2, or T3 and T4. Increasing bandwidth resulted in a decrease in the proportion of dry matter partitioning to the ear, but an increase to the leaves, reaching a significant level in T4 (Figure 4). The results show that an appropriate bandwidth can stabilize dry matter partitioning to the ear.

### 3.3. Differences in Ear Differentiation and Development

#### 3.3.1. Ear Differentiation Process

**Figure 5:**
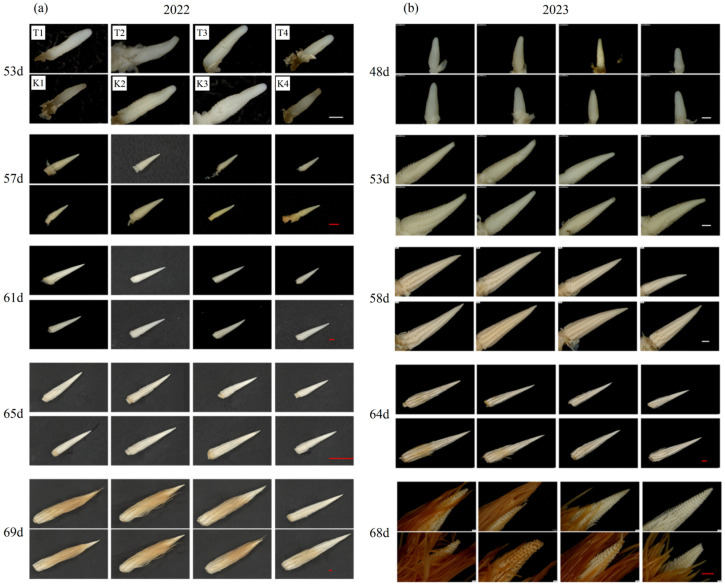
Differences in the young ear differentiation process between treatments in 2022 (**a**) and 2023 (**b**). The numbers on the left side of the figures represent the days after sowing, and the sub-figures are arranged in the order marked as 53 d after sowing in 2022 (**a**). The white line in the lower-right corner represents 500 μm, and the red line represents 2 mm.

Compared to K3 and K4, the differentiation process of young ears in T3 and T4 was delayed, and the difference appeared from 53 days and 48 days after sowing, i.e., the ear base developed during the spikelet primordium period. As ear differentiation progressed, the delay in differentiation continued. Compared to K1, the spikelet differentiation process of T1 was earlier in 2022 and was similar in 2023. Compared to K2, the spike differentiation of T2 in 2022 was slightly delayed from the spike primordium period differentiation and was similar in 2023. In 2022 and 2023, 57 days and 53 days after sowing, the ear base of each treatment differentiated into spikelet pair meristems, and the top differentiated into spikelet primordia. At 61 days and 53 days after sowing, the ear base entered the floret differentiation stage, and the top began to differentiate to form spikelet pair meristem. At 65 days and 64 days after sowing, the base differentiated into the late stage of floret differentiation, and the ovary began to expand and form silks. At 69 days and 68 days after sowing, the top of the young ear differentiated into the late stage of floret differentiation. The results show that there was no significant difference between T1 and T2 compared to K1 and K2, except that the process of ear differentiation was significantly delayed under T3 and T4 compared to K3 and K4 (Figure 5). Increasing bandwidth inhibited the ear differentiation and development.

#### 3.3.2. Ear Differentiation Characteristics

**Figure 6:**
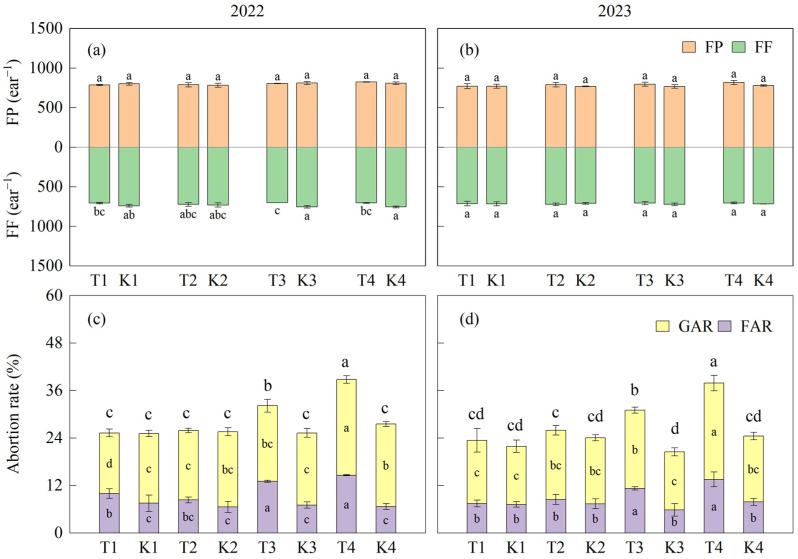
Differences in ear differentiation characteristics between treatments. (**a**,**b**) represent the total florets per ear and fertilized florets of maize in 2022 and 2023, respectively. (**c**,**d**) represent the floret abortion rate and grain abortion rate in 2022 and 2023, respectively. Different lowercase letters on the columns represent differences at the significant level (*p* < 0.05) within one index. Different lowercase letters above the columns in (**c**,**d**) represent differences in the total abortion rate. Values are means (n = 3), and bars indicate standard errors. FP, florets per ear, FF, fertilized florets, FAR, floret abortion rate, GAR, grain abortion rate.

There was no significant difference in total florets (FP) between treatments. In 2022, the fertilized florets (FF) in T3 and T4 was significantly decreased compared to K3 and K4, respectively. There was no significant difference in T1 and T2 compared to K1 and K2, respectively. However, in 2023, there was no significant difference in FF between treatments. Except for T1 and T2, the two-year floret abortion rate (FAR) and grain abortion rate (GAR) in T3 and T4 were significantly higher than in K3 and K4, respectively. The average values of FAR, GAR, and total abortion rate (TAR) in T3 were significantly increased by 89.0%, 19.9%, and 39.4%, respectively, compared to K3 over two years. FAR, GAR, and TAR increased by 95.4%, 31.1%, and 47.7%, respectively, in T4 compared to K4 (Figure 6).

**Figure 7:**
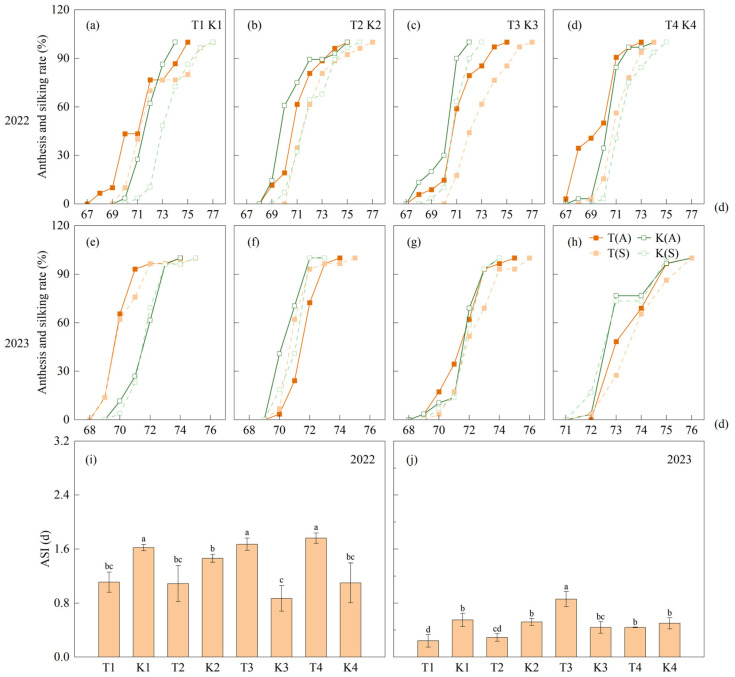
The proportion and interval of tasseling and silking of maize. (**a**–**d**) and (**e**–**h**) represent the proportion of tasseling and silking of maize intercropping and corresponding monoculture maize in 2022 and 2023, respectively, where (**a**) and (**e**), (**b**) and (**f**), (**c**) and (**g**), and (**d**) and (**h**) represent T1 and K1, T2 and K2, T3 and K3, and T4 and K4 treatments, respectively. (**i**) and (**j**) represent the interval between tasseling and silking in 2022 and 2023, respectively. Different lowercase letters represent significant differences (*p* < 0.05). Values are means (n = 3), and bars indicate standard errors. T(A), the proportion of tasseling in maize strip intercropping; K(A), the proportion of tasseling in monoculture; T(S), the proportion of silking in strip intercropping; K(S), the proportion of silking in monoculture; ASI, the interval between tasseling and silking.

Compared to the corresponding monoculture, the average overlap days of tasseling and silking in maize strip intercropping increased by 1.5 days, 1 day, 1 day, and 0 days over two years. Compared to K1, the tasseling and silking of T1 were 1.5 days and 1 day earlier, respectively. Tasseling of T2 and T3 was consistent with that of K2 and K3, while silking was delayed by 0.5 days and 1 day, respectively. Compared to K4, tasseling of T4 was 0.5 days earlier and silking was consistent. With increasing bandwidth, tasseling and silking were delayed, withT4 was delayed by 2 days compared to T2. Compared to the corresponding monoculture, the anthesis–silking stage (ASI) of T1 (0.68 days) and T2 (0.69 days) decreased by 0.4 days and 0.3 days, respectively, and T3 (1.27 days) and T4 (1.1 days) increased by 0.6 days and 0.3 days, respectively, over two years (Figure 7).

#### 3.3.3. Ear Length and Diameter

**Figure 8:**
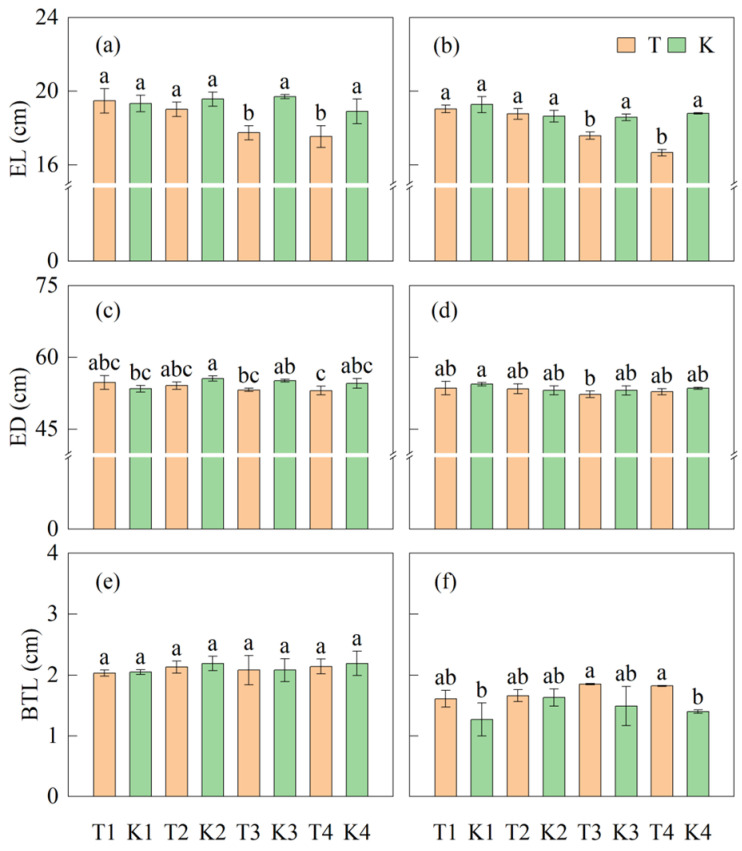
Differences in ear length (**a**,**b**), ear diameter (**c**,**d**), and barren tip length (**e**,**f**) at the maize maturity stage between treatments. (**a**,**c**,**e**) represent the ear characteristics in 2022, while (**b**,**d**,**f**) represent the ear characteristics in 2023. Different lowercase letters represent significant differences (*p* < 0.05).

There was no significant difference in ear length (EL) between T1 and T2 at the maize maturity stage compared to K1 and K2, respectively. Compared to K3 and K4, the EL in T3 and T4 was significantly reduced by 8.3% and 10.7%, respectively. No significant difference was found in ear diameter (ED) between intercropping maize and corresponding monoculture maize, nor between the bandwidths. Similar results also appeared on the barren tip length (BTL, Figure 8).

### 3.4. Yield and Components

No significant difference was observed in the number of production ears (NPE) between treatments over two years. Compared to the corresponding monoculture, grains per ear (GPE) in T1 and T2 did not significantly differ, whereas T3 and T4 exhibited a decrease of 9.6% and 10.2%, respectively. There was no significant difference in the 1000-grain weight (TGW) between T1 and K1 over the two years. T4, however, showed a significant decrease of 17.9% compared to K4. As bandwidth increased, GPE significantly decreased, with T4 experiencing an 11.5% reduction compared to T2 over two years. Compared to corresponding monoculture, maize yield (M) decreased by −1.7%, 5.2%, 18.5%, and 26.9%, respectively, with the yield in T3 and T4 reaching a significant level (*p* < 0.05) over the two years (Table 1).

### 3.5. Correlation Analysis of Yield and Its Limiting Factors

**Figure 9:**
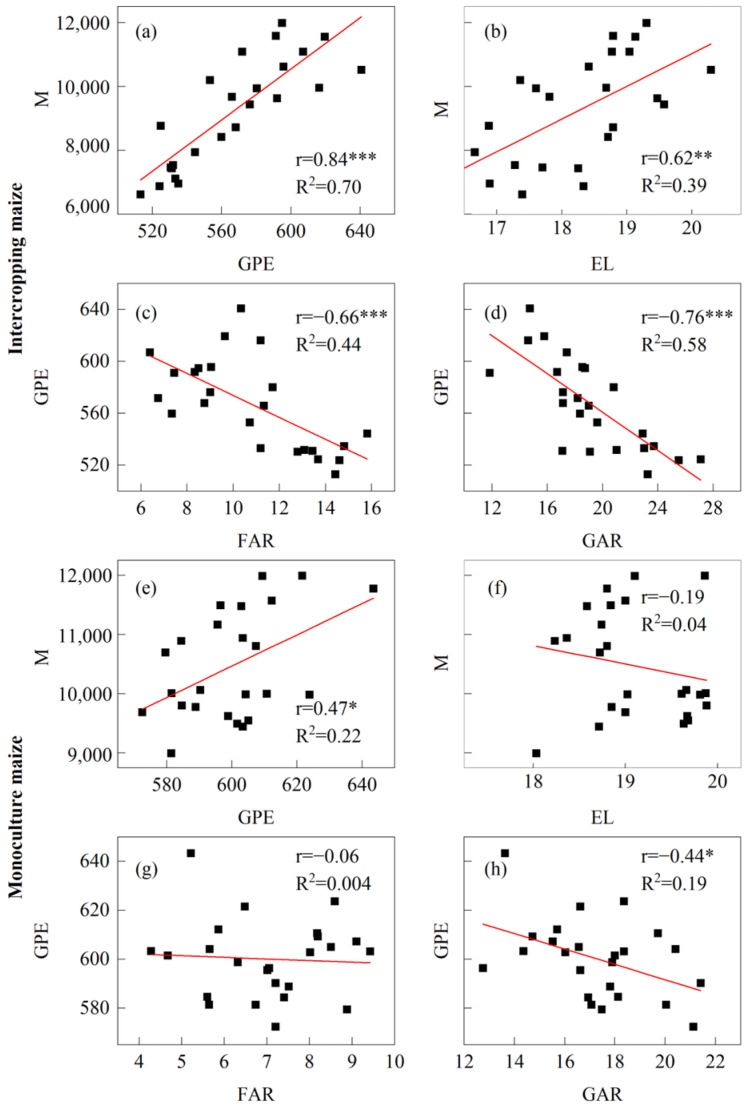
Correlation analysis of maize yield and its influencing factors. (**a**–**d**) and (**e**–**h**) represents the correlation analysis between maize yield and its influencing factors in maize strip intercropping and monoculture, respectively (n = 24). M, maize yield; EL, ear length at maturity stage; GPE, grains per ear; FAR, floret abortion rate; GAR, grain abortion rate. ***, *p* < 0.001, **, *p* < 0.01, *, *p* < 0.05.

Compared to corresponding monoculture maize, the correlation between maize yield (M) and grains per ear (GPE), as well as between M and ear length (EL), was enhanced. GPE in intercropping was significantly negatively correlated with floret abortion rate (FAR, r = −0.66 ***) and grain abortion rate (GAR, r = −0.76***). However, in monoculture maize, this correlation only existed between GPE and GAR (r = −0.44*). The results indicate that intercropping increased the sensitivity of floret differentiation, ear lengthening, and grain development (Figure 9).

## 4. Discussion

### 4.1. Appropriate Bandwidth Stable Dry Matter Partitioning to Ear

When crops sense the proximity of adjacent leaves or neighboring plants, their growth rapidly shifts towards the elongation of hypocotyls or stems. This response elevates the leaves, which serve as the primary photosynthetic organs, thereby reducing shading from neighboring plants through light-mediated signaling pathways. Simultaneously, to maximize light capture, crops avoid excessive leaf expansion, leading to thinner stems, and smaller and thinner leaves. Consequently, this morphological adjustment results in a loss of biomass and reduced yield [28,29,30,31]. Under conditions of intense light competition, plants prioritize enhancing their light-capture capacity by preferentially allocating resources to light-harvesting pigment proteins, thereby increasing the accumulation of light-harvesting pigments such as carotenoids [32,33,34,35]. However, this reallocation of resources results in a weakened photosynthetic electron transport capacity and a significant reduction in carbon anabolism proteins, including Rubisco enzymes, which in turn inhibits sugar accumulation [35,36,37,38]. Consequently, shading reduces the accumulation of sugars in leaves, increases the proportion of sugars allocated to leaves, and decreases the proportion allocated to grains, ultimately leading to yield loss [16,23,39,40]. In this study, as the bandwidth increased, the light transmittance of the ear layer of the wide row remained unchanged from the 2.4 m bandwidth, while the light transmittance of the ear layer of the narrow row decreased significantly, and the dry matter accumulation decreased, which may have been due to the reduced photosynthetic rate (Pn) of narrow-row leaves [4,12,13]. When the bandwidth increased to 2.8 m, the light transmittance of the narrow row decreased to 28.8%, and the growth and development of the leaves were inhibited [26,41]. In order to ensure the growth and development of narrow-row leaves and the ability of light capture, the proportion of dry matter partitioning to the leaves increased. This shift was driven by a reduction in the transport of dry matter from narrow-row leaves to the ear [4]. Also, the results at 2.8 m are consistent with conclusion under weak light in monoculture maize [39]. Although studies have shown that the photosynthetic rate of unshaded leaves increases significantly when unilateral light transmittance decreases [4], this conclusion excludes the influence of the underground part. As the bandwidth increases, the root development is inhibited, which in turn affects the photosynthetic performance of unshaded leaves [26]. Specifically, while unshaded leaves are positively regulated by shaded leaves [4], they are also constrained by limited underground nutrient availability [26]. Compared to monoculture maize, the light transmittance of wide and narrow rows at 1.6 m and 2 m decreased slightly. However, there was no significant difference in dry matter accumulation, with an abnormal value observed in 2020 attributed to higher rainfall. In addition to the positive regulation of Pn by enhanced light in wide rows, this effect may also be attributed to appropriate local weak light. This local weak light can improve the electron transport of shaded leaves and slow down the decrease rate of PEPC and Rubisco, thereby enhancing the Pn of shaded leaves [4]. Studies have shown that unilateral shading increases the transport of unshaded leaves to the ear [4]. In this study, there was no significant difference in ear dry weight between 1.6 m and 2 m bandwidths and the corresponding monoculture maize, which also confirmed this conclusion from the perspective of the ears. Appropriate bandwidth (1.6 m and 2 m) could improve the dry matter preferential partitioning to the leaves and stabilize the dry matter partitioning to the ear (Figure 4).

### 4.2. Intercropping Enhances the Sensitivity of Maize Floret Differentiation and Grain Development

Grain number per spike (GPE) significantly limited the yield potential of strip intercropping maize in this study (Table 1), which is consistent with previous studies on maize production under moderate stress conditions [39,42]. The larger bandwidth of strip intercropping inhibited the differentiation and development of ears (Figure 5, Figure 6, Figure 7 and Figure 8). The reason is that the dry matter accumulation decreased and the proportion of dry matter partitioning to the leaves increased, resulting in a decrease in the partitioning of the individuals to the ear. These results indicate that the dry matter accumulation per plant was affected by weak light as the bandwidth increased, and the dominant position of maize in the system was replaced by adjacent crops [26]. Previous studies have found that shading on both sides reduced dry matter accumulation, inhibited the differentiation and development of ears, and increased the anthesis–silking interval (ASI), affecting fertilization and leading to yield loss [19]. However, although the flowering and silking time of maize were affected by strip intercropping, there was no significant difference in the overlapping time of the full-bloom stage, and the ASI did not reach the threshold of significant effect on fertilization ability [23,27]. Strip intercropping maize can improve the asynchrony of flowering and silking under low light stress. Thus, GPE was less affected by the fertilization process in strip intercropping. The fertility of florets and ovaries is affected by the supply and utilization of assimilates [40,43]. Barren tip length was not a determinant factor influencing the variation in grains per ear in different bandwidths. This may because it is intricately linked to the regulatory network of genes and represents the outcome of long-term high-yield breeding objectives pursued by breeders [44]. Intercropping enhanced the correlation between GPE and yield, and enhanced the correlation between floret abortion rate and grain abortion rate and GPE. It can be seen that the floret differentiation, ear elongation, and ovaries of maize in strip intercropping were affected by weak light, but the total number of florets that could be genetically controlled [45] and the number of fertilized florets affected by the ASI were less affected by bandwidth. In this study, the lack of significant difference between 1.6 m and 2 m compared to the corresponding monoculture is attributed to the effective carbon compensation from wide-row leaves to narrow-row leaves under similar intraspecific competition [4,26]. The compensation ensured the supply of ear assimilates and stabilized both flower and grain abortion rates to ensure maize yield in intercropping. In this study, the narrow-row transmittance was maintained at no less than 35.2%, and could balance this compensatory effect and achieve a maize yield advantage. In maize-planting regions with abundant solar and thermal resources, expanding the bandwidth or increasing planting density can optimize maize strip intercropping practices.

## 5. Conclusions

As the bandwidth increased, dry matter accumulation decreased, while the partitioning ratio of assimilates to the leaves increased and that to ears decreased. This shift in resource allocation delayed the differentiation process of ears, and the lower anthesis–silking interval through synchronized tasseling and silking in intercropping did not affect fertilized florets. However, it led to an increased floret and grain abortion rate. Intercropping enhanced the sensitivity of floret differentiation, ear elongation, and grain development. The appropriate bandwidth—1.6 m and 2.0 m, with a unilateral light transmittance of not less than 35.2% in this study—had effective dry matter partitioning to the ear, which can meet the material supply during the differentiation and development of ears, reduce the floret and grain abortion rate, stabilize the grains per ear, and give full play to the advantage of maize yield in strip intercropping. Maize yield decreased by 12.8% and 24.9% at 2.4 m and 2.8 m. Therefore, the bandwidth in southwest China is 1.6 m and 2.0 m for intercropping systems. This study elucidates the critical periods limiting maize yield in intercropping from the perspective of ear development, thereby providing a theoretical basis for application of agronomic measures and the high-yield maize cultivation.

## Figures and Tables

**Table 1:** Yield and components of maize in intercropping and monoculture.

Year	Treatments	NPE ha^−1^	GPE ear^−1^	TGW g	M kg·ha^−1^
2022	T1	56,252.81 a	616.32 a	289.52 ab	10,037.45 a
	K1	55,558.33 a	603.30 ab	283.39 ab	9498.85 b
	T2	55,836.13 a	568.03 c	279.29 b	8863.392 c
	K2	56,175.65 a	604.22 ab	292.55 a	9923.72 ab
	T3	55,731.95 a	531.15 d	252.74 c	7481.48 d
	K3	56,237.38 a	597.00 ab	294.35 a	9877.86 ab
	T4	54,913.46 a	523.96 d	237.05 d	6821.01 e
	K4	56,574.11 a	581.37 bc	291.30 ab	9583.10 ab
2023	T1	59,080.33 a	597.73 a	327.34 a	11,553.25 ab
	K1	58,300.53 a	609.14 a	333.01 a	11,826.48 a
	T2	57,463.19 a	595.66 a	324.22 a	11,089.74 ab
	K2	58,706.64 a	592.08 ab	317.92 a	11,053.02 ab
	T3	57,141.3 a	566.41 b	307.3 a	9939.21 c
	K3	58,683.49 a	616.54 a	315.14 a	11,400.43 ab
	T4	56,499.42 a	534.12 c	263.70 b	7943.78 d
	K4	55,785.10 a	597.27 a	318.29 a	10,581.99 bc

Values are means (n = 3). Different lowercase letters represent significant differences (*p* < 0.05) among different treatments for maize within the same index in one year.

## Data Availability

Data are contained within the article.
